# 

**Published:** 1877-05

**Authors:** 


### CONTENTS.

COMMUNICATIONS.

Address—*Dr. A. T. Metcalf*.......... 185
Address—*Dr. R. G. Thomas*.......... 190
Transactions, Mississippi Valley Dental Association.......... 200
Transplantion.......... 214

SELECTIONS.

Quackery.......... 215
Physicial Benefit of Sunday.......... 217
Nervousness and Nervines.......... 218
Wood Rendered Incombustible.......... 219

EDITORIAL.

Who shall Enforce the Dental Law.......... 220
A New Head Rest.......... 221
Statement of a Case.......... 223
Transactions Ohio State Dental Society.......... 226
Canada Journal of Dental Science.......... 226
Ohio Dental College.......... 227
Michigan Dental Society.......... 227

